# Closing the Loop: A Software-Based Middleware Framework for Automated Vital Sign Integration With Cloud-Based Electronic Medical Records (EMRs)

**DOI:** 10.7759/cureus.90513

**Published:** 2025-08-19

**Authors:** Amy Avakian

**Affiliations:** 1 Diagnostic Radiology, Southern Hills Hospital and Medical Center, Las Vegas, USA

**Keywords:** electronic medical records (emr), healthcare software development, integrated software, software-based medicine, vital sign interpretation, vital sign monitoring

## Abstract

Despite significant advances in real-time physiologic monitoring, most hospitals continue to rely on manual vital sign documentation, a process prone to delays, transcription errors, and increased clinician workload. This paper presents a modular software-based middleware solution that enables seamless real-time transmission of vital signs from FDA-cleared monitoring devices to cloud-based electronic medical records (EMR). By leveraging Health Level Seven (HL7) and Fast Healthcare Interoperability Resources (FHIR) standards for interoperability, the system connects existing devices with major EMR platforms using standardized application programming interfaces, reducing infrastructure burden and improving documentation accuracy. We examine current barriers to adoption, including cost, workflow disruption, and compliance challenges, and highlight real-world examples of successful integration. Our analysis suggests that an interoperable and nurse-friendly architecture with built-in audit trails can enhance early warning system performance, reduce documentation errors, and alleviate clinician burnout by automating routine charting. This approach supports the transition to predictive, real-time clinical decision support in modern healthcare.

## Introduction

Vital signs guide almost every clinical decision, from recognizing early deterioration to escalating care. But in most hospitals, nurses are still writing down temperature, pulse, respiratory rate, blood pressure, and oxygen levels by hand, then entering them into the chart later. This slow and repetitive process creates delays, invites errors, and adds to an already heavy workload. We now have the technology to fix this. FDA-cleared monitors can automatically send vitals straight to the electronic medical record (EMR) in real time. Yet in practice, very few hospitals use them. The reasons are clear: high costs, clunky integrations, and workflows that are hard to change [1].

This paper looks at where things stand now. We review the available technologies, break down the main barriers to adoption, and propose a practical software solution that can link existing monitors with EMRs using standard application programming interfaces (APIs). The goal is less typing, fewer errors, and faster care powered by real-time data.

## Technical report

Review of current technologies

A growing number of FDA-cleared vital sign monitors now support real-time data transmission and integration with EMRs. These devices use wireless protocols such as Bluetooth, Wi-Fi, or cellular networks to transmit data either directly to EMRs or via cloud-based APIs. The systems vary in form factor, clinical use case, and depth of physiological monitoring, but all share the goal of automating documentation and enabling continuous patient tracking. Table 1 summarizes select FDA-cleared vital sign monitors with real-time EMR integration, highlighting their core features and transmission capabilities.

**Table 1:** Summary of FDA-Cleared Real-Time Vital Sign Monitors With EMR Integration Summary of FDA-cleared real-time vital sign monitoring devices compiled from publicly available manufacturer documentation and product specifications [1]. FDA: United States Food and Drug Administration; EMR: electronic medical record; BP: blood pressure; SpO_2_: peripheral capillary oxygen saturation; HR: heart rate; ECG: electrocardiogram; EKG: electrocardiography

Device/System	Manufacturer	Key Features	FDA Clearance	EMR Integration
VitalStream®	Caretaker Medical	Wireless, wearable monitor; cardiac output and BP; cloud-enabled	Yes	Yes
Portrait VSM	GE HealthCare	Wireless monitor; EMR integration	Yes	Yes
Connex VSM	Welch Allyn (Hillrom)	Real-time data upload; reduces manual entry	Yes	Yes
Vitals360®	VoCare	ECG, glucose, BP, SpO_2_; cloud-connected	Yes	Yes
BioButton®	BioIntelliSense	Continuous multi-parameter monitoring	Yes	Yes
Sempulse Halo	Sempulse	Rugged; cloud integration; emergency-ready	Yes	Yes
CardioWatch 287	Corsano Health	Wearable bracelet; real-time HR and respiration	Yes	Yes
QardioCore	Qardio	Ambulatory ECG/EKG monitor	Yes	Yes
CardiacSense CSF-3	CardiacSense Ltd.	Smartwatch with ECG, SpO_2_	Yes	Yes
EarlySense InSight	EarlySense	Contact-free continuous monitor	Yes	Yes

Barriers to adoption

Despite the availability of FDA-cleared systems that enable real-time vital sign transmission, several systemic and operational barriers continue to hinder widespread adoption across healthcare institutions.

Cost and infrastructure

Implementing fully integrated monitoring systems involves high initial expenses, including hardware procurement, software licensing, IT configuration, and staff training. These costs are particularly burdensome for community and rural hospitals, where budget constraints often prevent investment in new clinical technology [2]. Without demonstrable short-term return on investment, leadership may be reluctant to fund large-scale deployments.

Workflow resistance

Changes to established clinical routines often face resistance. Many nurses and clinicians remain skeptical of automation, viewing manual confirmation as more reliable. Even when automated systems are deployed, nursing staff are frequently required to recheck and manually input vital signs, which creates redundancy and undermines efficiency gains. This perceived lack of trust in automated readings slows adoption and limits workflow optimization [3].

Legacy EMR incompatibility

Many hospitals still operate on outdated or highly customized EMR platforms that are not compatible with modern monitoring systems. Bridging these technological gaps often requires third-party middleware or the development of expensive interface engines. Incompatibility with Health Level Seven (HL7) or Fast Healthcare Interoperability Resources (FHIR) standards remains a persistent barrier to seamless data integration [2].

Legal and compliance concerns

Institutional policies and medico-legal standards often mandate human verification of vital signs, even when data is transmitted automatically. Nurses may be obligated to manually re-enter values into the EMR or sign off on auto-uploaded data to satisfy audit and liability protocols. This creates documentation redundancy that neutralizes the benefits of real-time automation [3].

Data security and privacy

Cloud-connected devices must comply with the Health Insurance Portability and Accountability Act (HIPAA) and meet rigorous cybersecurity standards. Many hospitals are hesitant to rely on third-party or externally hosted systems to manage protected health information, particularly when the vendor cannot guarantee complete control over data storage, encryption, and breach response protocols [4].

These barriers contribute to the continued reliance on manual charting. Vital signs, including temperature, heart rate, respiratory rate, blood pressure, and oxygen saturation, are often still written on paper and later transcribed into the EMR. This delay not only burdens staff but also impairs the performance of early warning systems that depend on continuous and accurate data streams to detect patient deterioration. Research has shown that data latency and entry errors can significantly undermine the predictive value of clinical decision support tools [3].

Proposed solution: modular middleware platform

To address the gap between real-time vital sign monitoring and clinical documentation, we propose a plug-and-play middleware architecture designed for flexibility, scalability, and interoperability. The system is composed of five key components that together form a seamless pipeline from bedside monitoring to EMR documentation.

Universal Interface Layer

At the device level, a lightweight hardware or software agent connects to FDA-cleared vital sign monitors via USB, Bluetooth, or local area network (LAN). This interface continuously extracts physiological data, regardless of the manufacturer, and transmits it to the middleware for processing. The goal is to enable real-time data acquisition without requiring hospitals to replace their existing monitoring infrastructure.

EMR Adapter Engine

Captured data is converted into standardized formats using a mapping engine that supports HL7 version 2.5 and FHIR protocols. This ensures compatibility with a wide range of EMR systems and enables structured data to be pushed directly into patient records. Previous work has demonstrated the feasibility of HL7-based middleware for physiological data integration [5].

Validation and Patient Matching Layer

Each reading is associated with the correct patient using either QR code scanning or radio-frequency identification (RFID) badge systems. An optional verification screen allows nurses to confirm identity and values before upload. This layer also includes a basic rules engine that detects abnormal values and flags them for further review, ensuring that critical outliers are not overlooked.

Clinical Dashboard and Audit Logging

A secure web-based dashboard enables clinicians to view, validate, and confirm real-time vital signs from any device. This interface provides detailed audit trails, transmission logs, and alerts in case of connectivity loss or data discrepancies. It offers transparency and traceability, supporting both clinical decision-making and compliance requirements.

Security and Compliance Framework

The entire platform is designed with end-to-end encryption and robust access controls that comply with HIPAA regulations. Role-based user access, encrypted data transmission, and secure authentication protocols protect patient information throughout the data lifecycle. Middleware platforms that support overlay networks and real-time data security have been successfully piloted in mobile patient monitoring systems, demonstrating their feasibility in clinical environments [6].

This modular middleware platform is designed to be EMR-agnostic and compatible with leading vendors such as Epic, Cerner, and Meditech via Substitutable Medical Applications, Reusable Technologies (SMART) on FHIR protocols. It can scale from small community hospitals to large tertiary care centers without requiring major infrastructure changes. By streamlining data acquisition, standardizing integration, and maintaining compliance, this approach enables hospitals to unlock the full value of automated vital sign monitoring.

## Discussion

Benefits and impact

The adoption of a modular middleware system for real-time vital sign integration offers significant clinical and operational benefits. Automating the documentation process eliminates redundant manual entry, reducing transcription errors and improving the accuracy of patient records. Real-time data availability enhances clinical timeliness by enabling earlier recognition of deterioration and faster activation of protocols such as rapid response or sepsis bundles.

Beyond accuracy and speed, the use of structured, continuous data supports the implementation of predictive analytics. Systems trained on high-frequency physiologic data can power early warning scores, acuity prediction tools, and deterioration detection algorithms. Integrated monitoring dashboards can provide immediate clinical insight across multiple patients, supporting both inpatient care and remote monitoring models [7].

Operationally, the time savings are substantial. In a 150-bed hospital, eliminating manual vital sign entry could save over 8,000 hours of nursing time per year, assuming an average of two to three minutes saved per documentation event. This freed capacity can be redirected toward patient education, coordination of care, and clinical assessment, where human expertise is irreplaceable. Furthermore, the proposed platform is compatible with hospital-at-home and remote patient monitoring programs. Its ability to capture and transmit structured data in real time allows for seamless linkage between decentralized care settings and core EMR infrastructure [2].

The cost-efficiency of the system also deserves emphasis. Because it interfaces with existing FDA-cleared monitors and EMRs, the middleware avoids the need for full infrastructure replacement. Hospitals can achieve modern functionality with minimal hardware upgrades or vendor disruption, making this a scalable solution for both large urban medical centers and smaller community facilities.

Regulatory and ethical considerations

Any middleware platform that contributes to clinical decision-making may fall under the oversight of the FDA as a Class II medical device. If the system directly influences triage, diagnosis, or treatment decisions, such as by generating alerts, scoring acuity, or prioritizing care, its classification will depend on both its intended clinical function and technical capabilities. Developers must ensure that regulatory compliance is embedded from the outset, including device labeling, software validation, and clearly defined pathways for FDA clearance or exemption [7,8].

Data security is another foundational requirement. The platform must fully comply with HIPAA, incorporating secure authentication protocols, access controls, and encryption for both stored and transmitted data. For institutions with strict data custodianship requirements or limited trust in cloud infrastructure, options for local storage and on-premise deployment should be included. In an era of growing cyber threats and increasing digitization, the security architecture of any health information system must be designed with proactive risk mitigation and patient safety at its core [8].

Transparency and explainability are also critical for ethical implementation. Systems that automate data transmission or flag abnormal findings must provide clinicians with a clear understanding of how data is processed, scored, and presented. Clinicians must retain control, including the ability to override automated entries or alerts. Middleware tools must be auditable and traceable to ensure accountability in case of technical failure or clinical disagreement. Black-box systems that make decisions without visible logic undermine trust and are inappropriate for high-stakes environments such as acute care [8]. Ethical digital systems must not only function correctly but also be perceived as fair, understandable, and clinician-aligned.

Patient consent and data stewardship require equally careful design. Patients must be informed not only that their physiological data is being collected, but also how it will be used when contributing to machine learning development or being shared with commercial partners. Informed consent must go beyond a checkbox; it must be transparent, meaningful, and easy to revisit. Ethical frameworks for digital health must prioritize patient autonomy and clarity around data ownership, especially as the boundaries between clinical and consumer health technologies continue to blur [8].

As real-time, data-driven systems become more integrated into care delivery, regulatory and ethical frameworks must evolve accordingly. Middleware tools should be designed with security, oversight, transparency, and patient-centered ethics at their foundation. Only then can automation enhance clinical care while preserving the trust that is essential in every healthcare interaction.

## Conclusions

Real-time, cloud-connected vital sign monitoring is no longer a vision of the future. It is already possible, already cleared by regulatory agencies, and increasingly necessary. Yet despite the availability of these tools, adoption remains limited due to cost, workflow disruption, and resistance to change. A modular, software-based integration layer offers a flexible solution that connects existing monitoring devices to EMRs without the need for costly infrastructure replacement. Reimagining how vital signs are captured and transmitted is not just about efficiency. It is about creating clinical environments that are intelligent, adaptive, and driven by timely data. When vital signs flow into the electronic record in real time, they can power early intervention, support clinical prioritization, and improve outcomes. Structured data is the backbone of predictive systems such as early warning scores, deterioration detection, and acuity tracking. Automating documentation is the first step toward enabling these capabilities. The impact goes beyond the inpatient setting. As hospital-at-home programs and remote patient monitoring become more common, the ability to transmit real-time vital signs from the home becomes a critical part of modern care. Patients recovering from surgery, living with chronic heart failure, or undergoing chemotherapy can be safely monitored from home, reducing readmissions and improving quality of life. Middleware that supports this transition ensures continuity, oversight, and responsiveness across care settings.

Ethical and regulatory considerations must guide this transformation. Patients need to know how their data is being collected, stored, and used when it is used for training artificial intelligence or included in commercial development pipelines. Consent must be informed, and data governance must be transparent. Middleware software must be explainable and auditable. Clinical systems that make recommendations without transparency will not be acceptable in life-critical situations. Oversight features such as error reporting and clinician override must be built into every layer. Connecting vital sign monitors with electronic records through secure, standards-based software is not just a technical improvement. It is a requirement for safer, smarter, and more equitable healthcare.
